# “Strong Opinions, Loosely Held”: A Renewed Call for Error-driven Learning in Medical Education

**DOI:** 10.1007/s40670-026-02735-2

**Published:** 2026-04-22

**Authors:** Hunter Scott, Brian Devorkin

**Affiliations:** 1 Department of Internal Medicine, Cleveland Clinic, Cleveland, OH USA; 2 School of Medicine, Case Western Reserve University, Cleveland, OH USA

## Abstract

Improving clinical reasoning is a national priority to reduce medical error. This commentary advocates for “strong opinions, loosely held” as a cognitive aid to improve reasoning and retention in medical education. Learners should commit confidently to decisions while remaining open to revision as new evidence emerges. Grounded in prediction error theory, which emphasizes memory retention when expectations are upset, this approach mirrors expert clinician strategies. Case-based curricula can operationalize this by prompting early hypotheses and reassessment. Despite concerns about overconfidence, we view discomfort as natural when transitioning from passive to active learning, ultimately promoting autonomy, improved retention, and deeper engagement.

Sentiments on the act of learning medicine have been expressed and debated in various forms since the first physicians [1, 2]. Recently, optimizing the education of medical students, residents, and fellows has been the focus of many publications [3, 4]. In this commentary, we seek to further describe and promote the theory, utility, and practice in which learners develop a firm stance on the diagnosis and management of clinical cases (strong opinion) while remaining fluid in their ability to modify this perspective when confronted with information that alters their initial impression (loosely held). As the body of medical knowledge that trainees are expected to retain only continues to grow, we believe being able to improve memory retention using active learning is critical [5].

The U.S. National Academies of Sciences, Engineering, and Medicine’s seminal 2015 report on *Improving Diagnosis in Health Care* identified several pillars to improve healthcare quality and reduce diagnostic medical errors. Two of these recommendations relate to encouraging students to firmly commit to a diagnosis. The first establishes the need to “enhance health care professional training…in the diagnostic process,” specifically in areas such as clinical reasoning [6]. Second, and more importantly, the group noted an urgency to “[d]evelop and deploy approaches to identify, learn from, and reduce diagnostic errors and near misses in clinical practice,” including providing systematic feedback on diagnostic performance [6]. Building diagnostic reasoning requires active participation from learners, yet many current practices entail a trainee passively observing a masterful clinician approach a clinical problem, often without optimal active learning opportunities [7]. The literature offers support for medical students engaging with higher stakes on clinical problems as often as possible to build diagnostic reasoning capabilities and illness scripts [8–10]. The medical knowledge required for mastery continues to expand; thus, more efficient models of retaining information and improving active learning are crucial.

In this context, we define active learning as a structured engagement in which learners must commit to diagnostic reasoning, receive feedback, and revise their hypotheses; this process is supported by experiential and cognitive learning theory [11]. We argue the learning process works best when students take risks with flexible, robust sentiments that continue to evolve with support from the larger medical team. In the setting of overwhelming cognitive demands, and as a response to the desire of faculty, students, and patients for continuous improvement by medical professionals, we propose the aphorism “strong opinions, loosely held,” as a framework for learners and clinicians to meet this call to action.

The proposed heuristic, a simple guiding cognitive principle that assists in navigating complex diagnostic reasoning tasks under uncertainty, or “rule of thumb,” is a variant of a phrase attributed to Stanford University professor Paul Saffo, who first applied it to financial forecasting [12]. This idea differs from related concepts such as productive failure, desirable difficulties, and structured reflection. While these approaches also leverage challenge and reflection for learning, the “strong opinions, loosely held” phrase uniquely emphasizes real-time commitment to diagnostic hypotheses, followed by rapid evidence-based revision during the evolving clinical encounter. Critically, these two components are not in opposition to each other; rather, the strong opinions formed generate the expectation to make prediction error possible, while the loosely held nature allows for relative cognitive surprise when the initial hypothesis is altered by new data—this is not indecision.

The suggested heuristic is rooted in the neuropsychological concept of prediction error theory, which is best defined as “the mismatch between expected and current events” [13]. The “surprise” of getting an answer, or in medicine, the diagnosis and management of a clinical case, has been shown to be a key driver in how the brain can best retain information [14]. This practice improves retention, and we argue it is more enjoyable, albeit difficult at first. Improved learner engagement is also an intended side effect. In fact, this process on a different scale is employed by master clinicians who generate hypotheses and test them accordingly [14]. As an illustration of this concept, physicians frequently recount cases in which they made a diagnostic error and were surprised when this was discovered and corrected: an error they vow never to make again.

Given this evidence, we should reward learners not only for being correct, but also for taking on tough diagnostic and management challenges. We should celebrate when a complete, thoughtful approach is taken to reach an explicit medical conclusion by a trainee—even when the answer is wrong—if we want to prioritize the learning that leads to eventual reduction in diagnostic error. Overall, this would contribute to fostering a growth mindset and may be more enjoyable in the long term despite initial difficulty for learners and educators. Trainees, and medical students in particular, are best suited for this approach as the stakes are lower. The need for more efficient learning given the vast amount of required information is clear, but to implement this practice there must be a change within the greater academic medicine community to promote learner psychological safety.

A proposed framework for implementation in medical education follows: (1) Active commitment: learners articulate a clear diagnostic or management opinion based on available data; (2) Open-minded evaluation: trainees actively consider new information that may challenge their initial interpretation; (3) Evidence-based revision: learners continuously update their reasoning, reinforcing durable memory formation through prediction error and reflective adjustment. For example, during clinical rounds in a low health resource clinic serving patients from diverse backgrounds, learners may frequently encounter patients whose clinical presentations are shaped by multiple chronic conditions and social barriers to care. A patient presenting with shortness of breath may have poorly controlled diabetes, hypertension, asthma, as well as limited access to medications due to financial instability. A medical student may initially propose a leading diagnosis of acute asthma exacerbation with an accompanying management plan based on the history, but as additional information emerges (e.g., presence of jugular venous distension and orthopnea), the learner is encouraged to reassess and refine their reasoning. In navigating competing diagnostic possibilities and prioritizing urgent concerns, the learner must adapt their initial hypothesis while integrating the broader clinical and social context of the patient.

Challenges to our suggested viewpoint include a lack of psychological safety of learners at the bottom of the clinical hierarchy or concerns regarding overconfidence [15, 16]. Regardless, we acknowledge that due to the high oversight typical for novice learners, more senior clinicians should promote a safe environment for learning and subsequently encourage commitment to active learning through the “strong opinions, loosely held” approach [17]. The discomfort that some may experience using this approach to learn must be placed in context with the potential benefits: a more durable memory and more active involvement in clinical care [18]. 

Diagnostic error in this cognitive learning process only seeks to strengthen reasoning skills and foster robust topic memory [19]. However, it is likely some learners more accustomed to passive learning techniques may initially be uncomfortable with this method [20]. We advise that this discomfort is not only advantageous to a student’s learning process, but also a natural part of the transition from student to clinician. Moreover, embracing discomfort becomes a beneficial and essential skill later when taking the title of practitioner — no longer watching master clinicians, but instead starting to step into their shoes. While we acknowledge this approach is grounded in active learning and prediction error theory, no empirical assessments have been performed, though prospective studies are warranted to investigate potential benefits to learner engagement and knowledge retention.

To reduce untoward effects during implementation, we suggest that senior clinicians, accreditation bodies, medical schools, and graduate medical education training programs promote a “culture of safety” (a National Academies priority) regarding wrong clinical reasoning. Just as levels of autonomy vary by stage of competency and education, the level of risk tolerance with clinical reasoning should be bound by the same guardrails. Providing adequate debriefs and offering explanations to learners when applying our proposed lens is critical. For example, after giving structured space to practice clinical reasoning, educators should explain where learners were correct and where they were wrong in a supportive environment. 

We note that supportive environments in medical education are frequently defined in the literature, which can be used to aid integration of the framework [21]. This may foster short-term discomfort in exchange for long-term comfort in the process of active learning. One can easily apply our suggested heuristic to the development of case-based curricula, or simply employ it “on the fly” during clinical rounds in which learners might be asked to commit to the most likely diagnosis based on history alone and then reassess their defended position as more information (e.g., physical exam, laboratory studies, radiology) is presented. For those involved in graduate medical education, oral case presentations or formal sessions employing diagnostic reasoning are other suggested ideas with relatively low barriers toward implementation.

We argue that making a strong mental commitment to a diagnosis or treatment based on careful assessment of the patient mirrors our brain’s natural mechanisms for retaining information and is thus likely to improve diagnostic reasoning while encouraging more active participation in the clinical process. Practically, the simple aphorism “strong opinions, loosely held” we propose for use in medical education stipulates that not only do we learn best from independently forming reasoned clinical stances, but also that this requires the use of techniques to rapidly change our thinking if contradictory information refutes the original hypothesis [22]. Faculty can operationalize this framework by encouraging early hypothesis generation, fostering psychologically safe learning environments that normalize diagnostic uncertainty, and routinely revisiting initial clinical impressions as new evidence emerges. By developing strong medical opinions, and holding them loosely, medical students, residents, and fellows more effectively develop as practitioners, and pave the way toward a more innovative future of medical education outlined as a national priority. They will learn to seek out new information, be thought leaders, and challenge the status quo using clinical studies that provide evidence against once firmly held beliefs.

## Data Availability

Data sharing not applicable to this article as no data sets were generated or analyzed during the current study.
